# Smartphone Addiction and Checking Behaviors Predict Aggression: A Structural Equation Modeling Approach

**DOI:** 10.3390/ijerph182413020

**Published:** 2021-12-10

**Authors:** Shuna Shiann Khoo, Hwajin Yang

**Affiliations:** School of Social Sciences, Singapore Management University, Bras Basah, Singapore 178903, Singapore; shuna.khoo.2018@phdps.smu.edu.sg

**Keywords:** aggression, checking, objective smartphone use, problematic smartphone use, screen time, smartphone addiction

## Abstract

Despite the potential risks of excessive smartphone use for maladaptive outcomes, the link between smartphone use and aggression remains less understood. Furthermore, prior findings are inconclusive due to a narrow focus on limited aspects of smartphone use (e.g., screen time) and reliance on self-reported assessments of smartphone use. Therefore, using objective measures of smartphone use, we sought to examine the associations between several key indices of smartphone use—screen time, checking behaviors, and addictive tendency—and multifaceted aggression (i.e., confrontation, anger, and hostility). In a cross-sectional study, we administered a series of questionnaires assessing aggressive tendencies (i.e., The Aggression Questionnaire) and various aspects of smartphone use (*N* = 253, *M_age_* = 21.8 years, female = 73.2%). Using structural equation modeling, we found that smartphone checking and addictive smartphone use predicted only hostility. In contrast, both objective and subjective measures of screen time did not predict any facets of aggression. These results highlight differing impacts of various indices of smartphone use on aggression and imply that excessive checking and addictive smartphone use are problematic smartphone-use behaviors that require more targeted interventions with respect to hostility.

## 1. Introduction

Excessive and habitual smartphone use has been found to predict maladaptive outcomes, including emotional and physical problems (e.g., anxiety or sleep issues) [1]. However, relatively little research has focused on the relation between smartphone use and sociobehavioral outcomes. Excessive smartphone use adversely influences daily activities such as sleep quality [2], which in turn induces stress, frustration, and negative affect. Given that these negative emotions heighten one’s aggressive tendencies [3,4], it is plausible that excessive or addictive smartphone use may be related to aggression, which consists of instrumental (i.e., physical and verbal aggression), affective (i.e., anger) and cognitive (i.e., hostility) components [5]. As unmanaged aggression often leads to undesirable outcomes, it is crucial that we understand whether daily use of smartphones would unknowingly trigger one’s aggressive tendencies.

The frustration–aggression hypothesis [3,4] suggests that maladaptive smartphone use, which is associated with greater frustration and negative affect [6], could heighten aggressive inclinations. For instance, excessive smartphone use that induces online vigilance—a mindset of constant connectedness—causes stress and negative affect because of potential social pressure to be available all the time [7,8,9,10]. In a separate vein, excessive smartphone use has been shown to be associated with poorer sleep quality and shorter sleep duration [11,12], which implicate a loss of control over emotions and aggressive impulses [13]. In line with this notion, studies have found that problematic smartphone users experience sleep disturbance and display greater aggressive behaviors [2]. Therefore, maladaptive smartphone use would likely implicate heightened aggression.

Although a few studies have examined the relation between smartphone use and aggression, their results are inconclusive due to several limitations. First, previous studies have primarily examined aggression as a predictor of problematic or addictive smartphone use [14,15]. Second, they failed to consider diverse aspects of smartphone use (e.g., screen time, checking frequency, and addictive behavior) that likely heighten aggression [16]. Third, although aggression is a multifaceted construct with multiple components, including physical and verbal violence (instrumental/behavioral component), anger (affective/emotional component), and hostility (cognitive component) [5,17], previous studies have focused on limited aspects of aggression, such as anger [16], or treated aggression as a unidimensional construct by aggregating different components [15]. Lastly, previous studies have relied on generic and self-reported measures of smartphone use that are susceptible to memory bias and, thus, highlight the need for objective measures [18].

Given these existing studies and limitations, more research with conceptually refined constructs of smartphone use and rigorous methodology (e.g., objective assessment and latent variable analyses) is needed to precisely estimate the association between smartphone use and behavioral changes, especially aggression. To this end, we first aimed to clarify whether conceptually different constructs of smartphone use—screen time, checking behaviors, and addictive use—would similarly or dissimilarly influence aggression. The displacement–interference–complementary framework posits that varying aspects of smartphone use differently implicate psychological functioning, such as well-being [19]. For instance, the displacement hypothesis postulates that heavier smartphone use lowers well-being by displacing time spent on meaningful activities, while the interference hypothesis posits that smartphone checking behaviors induce poorer outcomes by interfering with individuals’ meaningful activities. Although these aspects of smartphone use are seldom clearly delineated, previous studies have shown that heavier smartphone use does not necessarily engender addictive use [20,21], and frequent checking alone is not indicative of problematic use [18]. Therefore, in view of these distinctive indices of smartphone use and their possibly disparate implications, it is vital that we examine how specific indices of smartphone use—screen time, checking behaviors, and addictive use—would influence individuals’ aggression.

Regarding smartphone screen time, the displacement hypothesis suggests that it could displace important activities such as sleep or in-person social interactions and lead to greater fatigue, loneliness, and anxiety [22,23], all of which likely trigger aggressive tendencies. Furthermore, the lack of meaningful activities (e.g., quality sleep, outdoor activities) that buffer against negative emotions may engender a vicious cycle that aggravates aggression. In support of this notion, large-scale studies have found that individuals with higher levels of screen time were more likely to lose their temper, were unable to switch tasks without anxiety or anger [24], and experienced poorer psychological well-being [25]. Thus, we expected that longer screen time (assessed objectively and subjectively) would heighten aggression.

Conversely, according to the interference hypothesis [19], frequent smartphone checking interferes with meaningful activities such as face-to-face interactions and results in frustration or heightened aggression. Notably, recent findings demonstrate that smartphone use is not detrimental to well-being when the environment does not implicate any social interaction [26]. Specifically, the study found that smartphone use in social settings interfered with the emotional benefits individuals could otherwise reap from their broader and richer social environment. Further, studies have shown that higher smartphone checking frequency predicted poorer cognitive control, which could adversely influence one’s impulsivity or aggression control [21,27]. Therefore, it is conceivable that frequent smartphone checking, rather than general use, may disrupt meaningful activities and magnify frustration and aggressive tendencies [19]. Accordingly, we hypothesized that smartphone checking would predict aggression.

Different from smartphone screen time and checking, smartphone addiction describes compulsive maladaptive habits with respect to smartphone use that impair daily living [1,28]. Individuals who struggle with smartphone addiction often miss planned work due to their smartphone use or feel anxious when they are unable to access their smartphones [29]. Importantly, a recent review of smartphone addiction elucidates its negative consequences, including control and emotional problems (e.g., anger, dysfunctional attitudes, and venting) [1]. Consistent with this notion, severe addictive smartphone use was found to be associated with greater worry and anger, which could culminate in aggression [16]. Other studies have found that smartphone addiction causes sleep disturbance [12], which affects emotional functioning and results in heightened aggression [2]. Given this, it is conceivable that addictive smartphone users may be easily provoked to become aggressive when their smartphone use is interfered with or when they encounter frustration. Therefore, we hypothesized that addictive smartphone use would predict aggression.

In short, we examined potentially different predictabilities of specific smartphone-use constructs (i.e., screen time, checking behavior, and addictive use) in aggressive tendencies—i.e., confrontation, anger, and hostility—which, respectively, correspond to behavioral, affective, and cognitive facets of aggression [17]. To this end, we employed rigorous structural equation modeling, which allows us to test the construct validity of aggression and provide a more precise estimate of the relation between smartphone use and aggression, while taking into account possible measurement error.

## 2. Methods

### 2.1. Participants

Two hundred and thirty-five university students who were smartphone users participated in the study (*M_age_* = 21.8 years, female = 73.2%; see Table 1 for details) in exchange for course credits and/or monetary means. However, due to technical errors, 20 participants’ objective smartphone use data were not collected; thus, analyses pertaining to objective smartphone use only involved 215 participants. All procedures were approved by the university’s institutional review board.

### 2.2. Measures

#### 2.2.1. Smartphone Screen Time

For objective smartphone use, participants provided screenshots of either the default iOS screen time application [30] or a specific Android screen time monitoring application [31]. Both captured the total amount of screen time in the previous week, which we then divided by the number of days to derive average daily objective smartphone use in hours.

For self-reported smartphone use, participants reported the estimated amount of time spent on 14 smartphone activities daily (e.g., text/instant messaging, email, social networking sites) on a 9-point scale (0 = *not at all*; 9 = *more than 10 h per day*) [32], and the number of hours across all activities were summed.

#### 2.2.2. Smartphone Checking

A three-item scale (α = 0.637) assessed smartphone checking behaviors [33]: (a) “In the past 7 days, on average, how often did you check your smartphone for new activity?” (1 = *only a few times a day*; 7 = *less than every 5 min*); (b) “In the past 7 days, on average, how often did you find yourself checking your smartphone when you had a few moments to spare, e.g., waiting at an elevator/stoplight/queue?” (1 = *almost never*; 5 = *almost always*); (c) “In the past 7 days, on average, how often did you find yourself checking your phone during conversations or when hanging around with friends?” (1 = *almost never*; 5 = *almost always*). Item responses were standardized and averaged for a single *Z*-score. Higher scores indicate more frequent smartphone checking.

#### 2.2.3. Smartphone Addiction

Smartphone addiction was assessed using a 10-item scale (α = 0.835; 1 = *strongly disagree*; 6 = *strongly agree*) [28]. Sample items include “Won’t be able to stand not having a smartphone” and “Missing planned work due to smartphone use.” Higher scores indicate more addictive use.

#### 2.2.4. Aggression

Participants’ aggression was assessed using an adapted version of The Aggression Questionnaire (1 = *does not describe me*; 5 = *describes me extremely well*) [5]. We used only three of the four subscales in the study: (a) 5-item confrontation (i.e., verbal aggression; α = 0.835; e.g., “I often find myself disagreeing with people”; (b) 7-item anger (α = 0.838; e.g., “I have trouble controlling my temper”); and (c) 8-item hostility (α = 0.882; e.g., “I am suspicious of overly friendly strangers”). The physical aggression subscale was not included because its items (e.g., “Given enough provocation, I may hit another person”; “There are people who pushed me so far that we came to blows”) are relatively male-specific, extreme, and infrequently observed among a sample of (healthy and non-psychopathological) college students in an everyday setting [34]. Higher scores indicate greater aggression.

#### 2.2.5. Covariates

We controlled for a host of key covariates including participants’ age, sex, monthly household income, and trait self-control, which have been shown to be associated with excessive smartphone use or aggression [6,35,36] (see Table 2 for Pearson correlations). Trait self-control was assessed using the 13-item Brief Self-Control Scale [37].

## 3. Results

### 3.1. Measurement Model of Aggression

**Figure 1:**
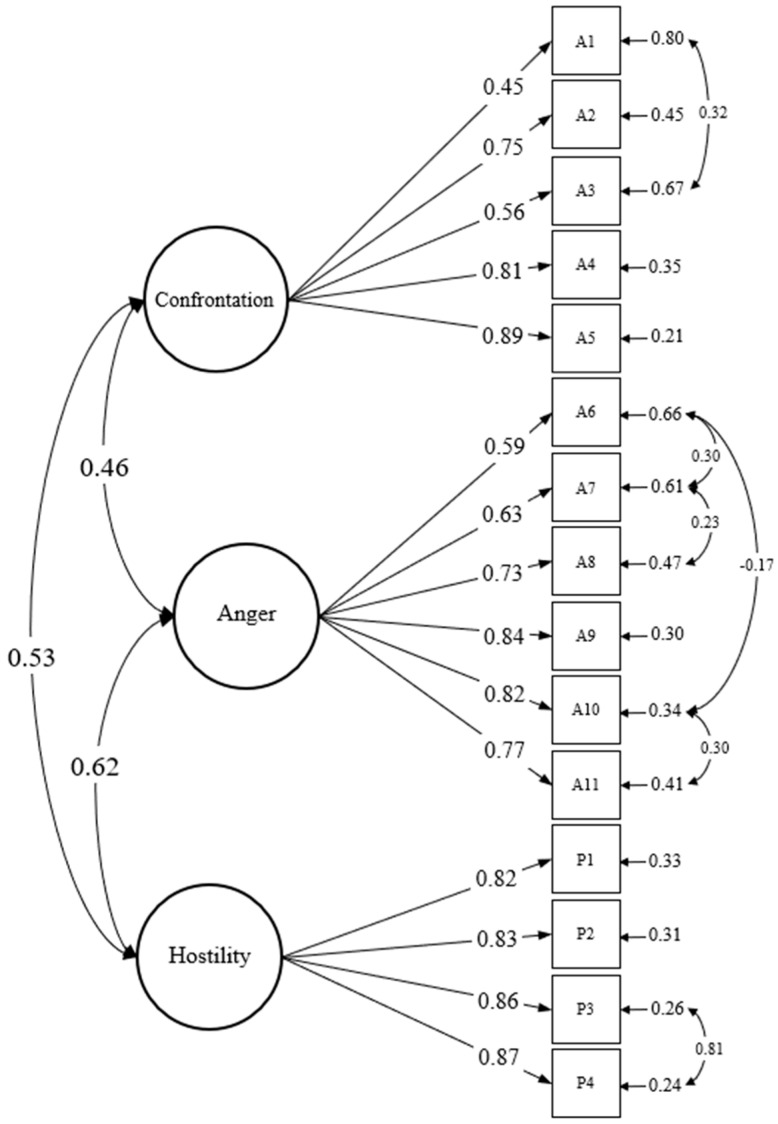
Full Measurement Model for Aggression Latent Factors. *Note.* Circles represent latent factors. Squares represent indicators; A1–A11 refer to scale items for confrontational and anger aggression. P1–P4 are parceled items of hostility. Values on the longer arrows signify path coefficients. Values for the shorter arrows represent residual variances. Double-headed curved arrows represent correlations between latent factors or residual variances. All coefficients shown are standardized and obtained statistical significance at the 0.05 level.

Model fit was assessed using Hu and Bentler’s (1999) fit criteria [38]. Specifically, the following standards were adopted: root mean square error of approximation (RMSEA) values equal to or below 0.08 and 0.06 for acceptable and good fit, respectively; comparative fit indices (CFI) and Tucker–Lewis index (TLI) close to or greater than 0.95; standardized root mean squared residual (SRMR) values equal to or below 0.08. When we fitted a three-factor model to the data—with scale items as indicators of the respective factors—the fit was unacceptable. Therefore, we (a) removed one redundant item, (b) parceled the indicators for hostility because parceling offers better psychometric properties [39], and (c) correlated residual variances of some similarly worded items (see Figure 1). The results show acceptable fit (see Table 3).

### 3.2. Structural Equation Models (SEM)

Using SEM, we regressed aggression (anger, confrontation, hostility) on each predictor—screen time, checking behavior, and addictive use—while controlling for important covariates (sex, age, monthly household income, and self-control). We found that smartphone checking significantly predicted hostility (β = 0.159, *SE* = 0.065, *p* = 0.014) but not anger (β = 0.074, *SE* = 0.069, *p* = 0.278) or confrontational tendencies (β = 0.002, *SE* = 0.069, *p* = 0.974). Similarly, smartphone addiction only predicted hostility (β = 0.171, *SE* = 0.069, *p* = 0.013) and not anger (β = −0.037, *SE* = 0.074, *p* = 0.618) or confrontation (β = −0.093, *SE* = 0.073, *p* = 0.203). These results provided partial support for our hypotheses. Contrary to our predictions, objective and subjective (i.e., self-reported) smartphone screen time did not predict any facet of aggression (all *p*s > 0.29; see Table 4 for more details).

## 4. Discussion

We found notable merits in assessing specific aspects of smartphone use, such as screen time, checking behaviors, and addictive use, because they are differentially related to aggression. Our findings, in part, support the frustration–aggression hypothesis. Specifically, smartphone checking and addictive use predicted the hostility facet of aggression, although smartphone screen time was not significantly associated with any component of aggression. There are two reasons why smartphone checking and addictive smartphone use evoke a more substantial level of negative affect and, consequently, aggression than a longer smartphone screen time. First, frequent smartphone checking and addictive smartphone use can be more immediately disruptive [40] than longer smartphone screen time and, thus, result in more negative affect, which in turn triggers aggression. Second, longer screen time can be attributed to productivity or social use, which may not necessarily entail greater frustration or negative affect that predispose aggression. In line with these notions, one study found that screen time was not associated with depression or anxiety severity, but frequency of screen unlocking was negatively associated to negative affectivity [41].

In addition, prior findings have shown that smartphone addiction and smartphone checking, especially phubbing (i.e., the act of snubbing someone in a social setting by concentrating on one’s smartphone), are driven by insecurity, such as the need for excessive reassurance [14] and heightened fear of missing out (FOMO)—i.e., fear that exciting things are happening without them [42,43]. Addictive use and smartphone checking could further exacerbate these behaviors and trigger hostile cognitive evaluations, such as heightened jealousy, resentment over unfair treatment, or suspicion that friends are gossiping behind their backs [17]. Given that addictive use and frequent smartphone checking likely disrupt daily living, these findings provide support for the interference hypothesis, in that smartphone uses that interfere with concurrent activities are the ones that cause maladaptive outcomes for individuals [19].

Notably, neither addictive use nor smartphone checking predicted affective (i.e., getting angry) or behavioral aspects of aggression (i.e., being confrontational by openly expressing one’s opposition). This provides preliminary evidence that the impacts of smartphone addiction and excessive checking may be restricted to cognitive evaluations—i.e., hostility. Although heightened hostility may be less immediately threatening than anger or confrontation, it can be more dangerous because it likely culminates in deeply seated cognitive evaluations that lead to poorer mental well-being such as depression [44]. Therefore, smartphone use that interferes with daily functioning, such as addictive use or excessive checking, should be carefully monitored and actively corrected to avoid longer-term mental health issues.

In contrast, objective and subjective assessment of smartphone screen time did not predict aggression. These null findings fail to support the frustration–aggression hypothesis and displacement hypothesis, suggesting that longer smartphone screen time does not necessarily have detrimental aggressive outcomes. Given that various motivations underlie smartphone use (e.g., productivity, addiction, or social purposes), it is conceivable that our participants’ heavy dependence on smartphones for possible academic or social purposes may not lead to maladaptive outcomes. Future studies that further investigate whether smartphone screen time influences individual outcomes could focus on time spent on specific smartphone activities—e.g., passive browsing versus active conversations—to gain a more nuanced understanding of the possible relations.

Our study is not without limitations. First, our smartphone checking items may not have assessed the checking phenomenon holistically because our items could not capture the contextual factors during checking (e.g., phubbing), which possibly explains its relatively lower reliability. Future research should employ more sophisticated experience sampling methods to mitigate this issue. Second, our study cannot confirm causality because of its cross-sectional and correlational nature. Third, given that our sample is relatively homogenous, small, and mostly females, caution is necessary when generalizing our results to other populations (e.g., middle-aged adults), who may have different smartphone-use patterns. Notwithstanding the limitations, however, our study provides crucial insights into how distinctive constructs of smartphone use are related to various facets of aggression.

## 5. Conclusions

Our study elucidates the link between smartphone use and aggressive behaviors, which has received relatively little attention in the literature, and provides important implications. Given our finding that different indices of smartphone use asymmetrically predict hostility in particular, it is important to note the distinctive predictability of the various indices of smartphone use. Therefore, future studies should adopt a multi-indices approach to more accurately delineate the relations between smartphone use and sociobehavioral outcomes. Additionally, more studies are needed to test the theoretical framework of the frustration–aggression hypothesis in relation to smartphone use by examining possible mediators (e.g., negative affect or frustration) between smartphone use and aggression. Our findings also imply that excessive smartphone checking and addictive use are the critical locus for interventions that can circumvent hostility by adjusting smartphone-use patterns in young adults. Importantly, given that both objective and subjective screen time measures did not predict any facet of aggression, it is critical to understand that restricting smartphone screen time alone may not be an effective strategy for curtailing aggressive tendencies.

## Figures and Tables

**Table 1:** Descriptive Statistics of All Variables and Covariates.

.	*M*	*SD*	Min	Max	Skewness	Kurtosis	Reliability ^1^
**Focal predictors**							
Objective screen time ^2^	5.37	2.18	0.94	12.82	0.58	0.19	−
Subjective screen time ^3^	15.86	8.13	3.50	55.50	1.56	3.72	−
Smartphone checking (Z-score)	0.00	0.761	−2.15	1.88	−0.06	−0.13	0.637
Smartphone addiction	3.03	0.88	1.00	5.20	0.21	−0.33	0.835
**Outcome variable: Aggression**							
Confrontation	2.37	0.82	1.00	4.86	0.64	−0.26	0.835
Anger	2.34	0.87	1.00	4.80	0.51	−0.15	0.838
Hostility	2.24	0.87	1.00	5.00	0.79	0.49	0.882
**Covariates**							
Age	21.79	1.72	18.00	27.00	0.16	−0.08	−
Sex ^4^	1.73	0.44	−	−	−1.05	−0.90	−
Monthly household income ^5^	4.17	2.33	1.00	9.00	0.71	−0.43	−
Self-control	3.01	0.64	1.46	4.62	−0.05	−0.38	−

*Note.*^1^ Reliability estimates were computed based on Cronbach’s alpha using all scale (subscale) items.;^2^ Due to technical errors, only 215 individuals’ objective screen time was collected.;^3^ One participant’s self-reported screen time (69.50 h) was removed because it was identified as an outlier. The removal of this outlier did not change results pertaining to self-reported smartphone use; ^4^ 1 = *Male*; 2 = *Female*; ^5^ 1 = Less than USD 2500; 2 = USD 2500–USD 5000; 3 = USD 5000–USD 7999; 4 = USD 7500–USD 9999; 5 = USD 10,000–USD 12,499; 6 = USD 12,500–USD 14,999; 7 = USD 15,000–USD 17,499; 8 = USD 17,500–USD 19,999; 9 = More than USD 20,000.

**Table 2:** Pearson Correlations between Variables.

	1.	2.	3.	4.	5.	6.	7.	8.	9.	10.
1. Confrontation	-									
2. Anger	**0.64**	-								
3. Hostility	**0.37**	**0.72**	-							
4. Objective screen time	0.05	−0.04	0.07	-						
5. Subjective screen time	−0.02	0.12	0.11	**0.31**	-					
6. Smartphone checking	0.04	**0.15**	**0.23**	**0.25**	**0.17**	-				
7. Smartphone addiction	−0.05	0.08	**0.25**	**0.26**	**0.19**	**0.44**	-			
8. Self-control	**−0.22**	**−0.29**	**−0.33**	**−0.26**	**−0.14**	**−0.22**	**−0.32**	-		
9. Age	**0.15**	−0.05	0.00	−0.11	−0.07	−0.08	-0.27	0.06	-	
10. Sex ^1^	**−0.25**	−0.02	**−0.15**	-0.04	0.09	0.03	0.08	0.12	**−0.45**	-
11. Monthly household income	−0.06	−0.12	−0.11	0.05	−0.09	−0.09	−0.02	0.04	**−0.21**	0.09

*Note.* Significant statistics at *p* < 0.05 level appear in bold; ^1^ Sex was coded as 1 = *Male*, 2 = *Female*.

**Table 3:** Model Fit Indices for Measurement and Structural Models.

	χ^2^	*df*	RMSEA	CFI	TLI	SRMR
**Measurement models**						
Three-factor aggression	561.56 ***	167	0.100	0.84	0.82	0.073
Three-factor aggression (modified) ^1^	168.86 ***	81	0.059	0.96	0.95	0.059
**Full measurement models**						
Objective screen time	185.41 ***	97	0.062	0.96	0.95	0.058
Subjective screen time	185.25 ***	97	0.062	0.96	0.95	0.061
Smartphone checking	187.70 ***	97	0.063	0.96	0.95	0.068
Smartphone addiction	205.91 ***	97	0.067	0.95	0.94	0.069
**Adjusted structural models ^2^**						
Objective screen time	223.98 ***	142	0.052	0.96	0.94	0.052
Subjective screen time	239.35 ***	142	0.054	0.96	0.94	0.052
Smartphone checking	230.22 ***	142	0.051	0.96	0.95	0.051
Smartphone addiction	239.54 ***	142	0.054	0.96	0.94	0.053

*Note.* *** *p* < 0.001; ^1^ The modified model included the removal of one redundant item from the anger subscale, parceling items from the hostility scale, and correlating the residual variances of some similarly worded items; ^2^ Adjusted models included the covariates of age, sex, monthly household income, and self-control.

**Table 4:** Path Coefficients for Adjusted Structural Models with Covariates.

	Confrontation			Anger			Hostility		
Predictors	β	*SE*	*p*	β	*SE*	*p*	β	*SE*	*p*
Objective screen time	0.018	0.073	0.804	−0.107	0.073	0.141	−0.014	0.070	0.837
Subjective screen time	−0.021	0.068	0.762	0.069	0.068	0.311	0.070	0.066	0.288
Smartphone checking	0.002	0.069	0.974	0.074	0.069	0.278	**0.159**	**0.065**	**0.014**
Smartphone addiction	−0.093	0.073	0.203	−0.037	0.074	0.618	**0.171**	**0.069**	**0.013**

*Note.* Significant statistics appear in bold.

## Data Availability

The dataset, material, and code are available from the corresponding author upon request.
